# PLA1A2 platelet polymorphism predicts mortality in prediabetic subjects of the population based KORA S4-Cohort

**DOI:** 10.1186/1475-2840-13-90

**Published:** 2014-05-05

**Authors:** Bernd Stratmann, Tao Xu, Christa Meisinger, Barbara Menart, Michael Roden, Christian Herder, Harald Grallert, Annette Peters, Wolfgang Koenig, Thomas Illig, Heinz-Erich Wichmann, Rui Wang-Sattler, Wolfgang Rathmann, Diethelm Tschoepe

**Affiliations:** 1 Heart and Diabetes Center NRW, Ruhr University Bochum, Georgstr. 11, D-32545 Bad Oeynhausen, Germany; 2 Research Unit of Molecular Epidemiology, Helmholtz Zentrum Muenchen, German Research Center of Environmental Health, Neuherberg, Germany; 3 Institute of Epidemiology II, Helmholtz Zentrum Muenchen, German Research Center of Environmental Health, Neuherberg, Germany; 4 Institute for Clinical Diabetology, German Diabetes Center, Leibniz Center for Diabetes Research at Heinrich Heine University, Duesseldorf, Germany; 5 Department Internal Medicine II, University Clinic Ulm, Ulm, Germany; 6 Hannover Unified Biobank, Hannover Medical School, Hannover, Germany; 7 Institute of Epidemiology I, Helmholtz Zentrum Muenchen, German Research Center of Environmental Health, Neuherberg, Germany; 8 Institute of Medical Informatics, Biometry and Epidemiology, Ludwig-Maximilians-Universität, Munich, Germany; 9 Institute of Biometrics and Epidemiology, German Diabetes Center, Leibniz Center for Diabetes Research at Heinrich Heine University, Duesseldorf, Germany

**Keywords:** Glycated hemoglobin, Platelet glycoprotein receptor polymorphism, Mean platelet volume, All-cause mortality, Glycemic management, Epidemiology

## Abstract

**Objective:**

The genetic polymorphism concerning the ß3-subunit of platelet integrin receptor glycoprotein IIIa is held responsible for enhanced binding of adhesive proteins resulting in increased thrombogenic potential. Whether it is associated with mortality, HbA1c or platelet volume is tested prospectively in an epidemiological cohort.

**Research design and methods:**

Population-based Cooperative Health Research in the Region of Augsburg (KORA) S4-Survey (N = 4,028) was investigated for prognostic value of PLA1A2-polymorphism regarding all-cause mortality, correlation with HbA_1c_, and mean platelet volume. Multivariate analysis was performed to investigate association between genotype and key variables.

**Results:**

Prevalence of thrombogenic allele variant PLA2 was 15.0%. Multivariate analysis revealed no association between PLA1A2 polymorphism and mortality in the KORA-cohort. HbA_1c_ was a prognostic marker of mortality in non-diabetic persons resulting in J-shaped risk curve with dip at HbA_1c_ = 5.5% (37 mmol/mol), confirming previous findings regarding aged KORA-S4 participants (55–75 years). PLA1A2 was significantly associated with elevated HbA_1c_ levels in diabetic patients (N = 209) and reduced mean platelet volume in general population. In non-diabetic participants (N = 3,819), carriers of PLA2 allele variant presenting with HbA_1c_ > 5.5% (37 mmol/mol) showed higher relative risk of mortality with increasing HbA_1c_.

**Conclusion:**

PLA1A2 polymorphism is associated with mortality in participants with HbA_1c_ ranging from 5.5% (37 mmol/mol) to 6.5% (48 mmol/mol). Maintenance of euglycemic control and antiplatelet therapy are therefore regarded as effective primary prevention in this group.

## Background

Platelets play an important role in primary hemostasis and are involved in atherosclerosis and atherothrombotic events. Inhibition of platelet aggregation is the key step of any treatment of vascular disease. Metabolic conditions like hyperglycemia influence platelet reactivity and the response to platelet inhibitors through direct effects and by glycation of platelet proteins, especially in type 2 diabetes. Increased platelet reactivity involves intensified adhesion and aggregation in patients with diabetes mellitus or those at high risk for the disease [1]. A subpopulation of large, hyperactive platelets circulates in these patients, at a level similar to that predicted from the results of patients who have experienced myocardial infarction [2]. This suggests that the elevated potential for aggregation of such platelets lowers their threshold for activation, thus contributing to the increased incidence of acute cardiovascular events in diabetes mellitus. As a determinant of platelet activation mean platelet volume (MPV) is an emerging risk factor for atherothrombosis [3]. The increase in MPV may precede acute myocardial ischemia, acute myocardial infarction, coronary atherosclerosis, the presence and the short-term prognosis and the long-term risk of stroke and thus is in association with major cardiovascular events [4]. Case–control studies have documented significant positive associations of MPV with type 2 diabetes mellitus [5], pre-diabetes, obesity, and other metabolic risk factors, whereas smaller platelets are detected in chronic inflammatory disease, inflammatory bowel disease or rheumatoid arthritis [6]. The platelet count is inversely related to the MPV, patients with low MPV present with higher numbers of platelets and vice versa [7-9].

In a study on Japanese subjects MPV in patients with prediabetes was shown to be higher if compared to normal subjects, and it was positively associated with levels of fasting blood glucose in prediabetic and normal subjects [10]. In a Korean study MPV had a significant positive relationship with FPG after adjusting for diabetes in women as a confounding factor pointing out a gender specifity. The positive relationship between an increased glucose level and increased MPV was shown to be a unique phenomenon of diabetes itself. [11]. MPV values seem to be influenced by antidiabetic therapy, MPV is increased in patients with uncontrolled type 2 diabetes mellitus, and was significantly higher in diabetic patients treated with oral hypoglycemic therapy than in those patients on insulin therapy [12].

Numerous polymorphic surface glycoprotein receptors are responsible for platelet functionality with membrane glycoprotein (GP) IIb/IIIa playing a major role in platelet function. It enables stimulated platelets to bind to fibrinogen and related adhesive proteins, a process that is considered central in the development of thrombosis. The gene encoding GPIIIa shows a common platelet antigen polymorphism [PLA1A2, (*ITGB3* rs5918)] at position 1565 in exon 2 of the coding region for glycoprotein IIIa and results in a leucine-proline exchange [13]. The presence of the PLA2 allele was first reported in 1996 to be associated with an increased risk of coronary heart disease (CHD) [14]. The importance of the GPIIb/IIIa receptor has been further supported by clinical trials in which GPIIb/IIIa antagonists have been shown to reduce restenosis rate after angioplasty and to reduce the morbidity and mortality associated with unstable angina, high-risk coronary angioplasty, and acute myocardial infarction [15]. Studies on the PLA1A2-polymorphism and coronary risk suggest an influence of the PLA2 allele on the clinical phenotype and the interaction with other environmental factors [16]. The hyperaggregability associated with the PLA2 allele has been linked to an increased surface expression of GPIIb/IIIa receptors and increased affinity for fibrinogen [17]. The result of this altered expression is discussed controversially; because some studies suggest an association of the PLA2 allele with a greater risk of coronary events others do not support this assumption [18,19]. In particular, the strongest effect of the PLA2 allele was expressed on the risk of occlusion after revascularization procedures, mainly after stent implantation [20]. Some more recently published analyses do not support this hypothesis [21]. Hyperresponsiveness to agonists has been demonstrated in platelets positive for the PLA2 allele *in vitro*[16,17]. In a mechanism possibly unrelated to its effect on platelet reactivity to aggregating stimuli, the presence of the PLA2 allele might influence the antiaggregatory effect of platelet inhibitory drugs such as acetylsalicylic acid (ASA), clopidogrel, and GPIIb/IIIa antagonists [17]. Studies evaluating healthy donors indicate a possible role of the PLA2 allele in ASA resistance based on measures of platelet function, particularly in patients homozygous for PLA2 [22].

Beside the inconsistent reports on the predictability of the PLA polymorphism on cardiovascular events Tschoepe and coworkers found a significant association with the metabolic condition of type 2 diabetes mellitus in an analysis of 112 consecutive patients additionally classified according to the presence of macrovascular disease published earlier [23]. This finding is in contrast to a later publication of Maerz and coworkers from the Ludwigshafen Risk and Cardiovascular Health Study which revealed no association of the GPIIIa PLA1A2 polymorphism with type 2 diabetes, glucose metabolism, angiographically proven CHD or myocardial infarction [24].

With this regard, the aim of this prospective analysis of the KORA S4-survey is to clarify 1) the predictive role of the PLA1A2 polymorphism in the general population in terms of all-cause mortality, 2) its relation with HbA_1c_, and 3) its relation with main characteristics of platelet morphology.

### Research design and methods

#### Study population

The KORA study region consists of the city of Augsburg and the two surrounding districts with about 600,000 inhabitants in 1999. The Bavarian ethic committee approved the KORA S4 study (conducted between 1999 and 2001) which followed the declaration of Helsinki; informed consent was given by each participant. The initial study sample involved 6,640 subjects randomly drawn from the general population. Altogether 4,261 subjects participated in the baseline study (response 67%). Of those, 4,028 had been characterized according their PLA1A2 polymorphism by a flow cytometry based assay as described elsewhere [25] and could be included in the present analysis. Briefly, the polymorphism was determined from frozen EDTA cell samples by flow cytometry analysis using the stereospecific monoclonal antibody SZ21 directed against the ß3-subunit of the GPIIb/IIIa receptor. Mortality was followed up for a maximum of 10 years and cause of death was coded using common ICD coding. Blood collection and processing was described earlier [26]. Diabetes was defined based on self-reported physician diagnosis, use of antidiabetic agents and/or HbA_1c_ levels at baseline ≥6.5% (48 mmol/mol) (N = 209 participants) [27]. HbA_1c_ was determined centrally at baseline [26]. HbA1c-values were determined using a turbidimetric immunologic assay (Tina-quant, Roche Diagnostics). The interassay coefficients of variation were 3.9% at HbA1c 5.7% (39 mmol/mol) and 5.2% at HbA1c 9.7% (83 mmol/mol).

Descriptive analysis results of the population characteristics were reported as mean ± standard deviation (SD). Comparison between the groups was done by Mann–Whitney testing or one-way ANOVA followed by Dunnett’s multiple comparison post-test for continuous data and Fisher’s exact test for categorical data.

A multivariate logistical regression model was used to evaluate the cross-sectional association of genotype with HbA_1c_, MPV, platelet mass and platelet count. Variables investigated for possible confounding included age, sex, BMI, waist-hip ratio, diastolic and systolic blood pressure, cholesterol levels (total, HDL, and LDL), smoking status (categorized: non-smoker, former smoker, current smoker), high alcohol intake (categorized: ≥20 g/day for women; ≥40 g/day for men), leisure time physical activity (categorized: >1 h per week). Association between platelet count and covariates were investigated by linear regression model.

Cox proportional hazards model was used for a multivariate analysis of the risk of overall death with genotypes, HbA_1c_ level and platelet morphology (MPV, platelet count, and platelet mass), taking the same adjustment as previously described. Statistical analysis was done using R version 2.15.1 (The R Foundation for Statistical Computing). *P* values <0.05 were regarded statistically significant.

## Results

### Population characteristics and genotype distribution

4,261 subject data were available for evaluation from the KORA S4-survey. 4,028 subjects had been characterized according to their HbA_1c_, PLA1A2 genotype was determined by flow cytometry and data on the survival status were available. Total prevalence of the PLA2 allele was 15.0%, genotype distribution was as follows: A1A1: 2,912/4,028 = 72.3%, A1A2: 1,027/4,028 = 25.5%, A2A2: 89/4,028 = 2.2%. For evaluating the role of the PLA1A2 genotype two groups were set up comprising A1A1 genotypes and A1A2/A2A2 genotypes, called AxA2. We detected no significant difference in prevalence of AxA2 genotype in the living and deceased participants (Table 1). The other variables which differed between living and deceased people, such as diabetes prevalence, age, BMI, were taken as covariates in the following multivariate analysis.

**Table 1:** Population characteristics

	**Surviving persons**	**Deceased persons**	***P***
N [subjects]	3,789	239	
No. of diabetic subjects	156 (4.1%)	53 (22.2%)	<0.0001
GenotypeAxA2 [%]	27.6%	30.1%	>0.05
Age [years]	48.2 ± 13.7	63.3 ± 9.7	<0.0001
	range : 54–75 years	range : 35–75 years	
BMI [kg/m^2^]	27.1 ± 4.6	28.6 ± 4.7	<0.0001
Waist to hip-ratio	0.864 ± 0.088	0.932 ± 0.083	<0.0001
Blood pressure diastolic [mm Hg]	80.3 ± 10.4	80.9 ± 11.3	>0.05
Blood pressure systolic [mm Hg]	127.7 ± 19.0	139.6 ± 22.1	<0.0001
HbA_1c_ [%]	5.55 ± 0.58	5.90 ± 0.95	<0.0001
HbA_1c_ [mmol/mol]	37.2 ± 6.3	41.0 ± 10.4	
FBG [mg/dl]	106.6 ± 33.1	118.0 ± 42.9	<0.0001
	(1,428 subjects)	(201 subjects)	
Total cholesterol [mg/dl]	226.7 ± 43.3	235.6 ± 49.5	0.0014
HDL cholesterol [mg/dl]	57.8 ± 17.0	55.9 ± 16.8	>0.05
LDL cholesterol [mg/dl]	136.6 ± 41.4	145.7 ± 42.9	0.0012
Mean platelet volume [fl]	8.722 ± 0.94	8.834 ± 1.066	0.061
Platelet count [/nl]	244.4 ± 57.3	220.9 ± 65.4	0.0001
Platelet mass	2,108 ± 450	1,924 ± 519	0.0001

Analysis results of the population characteristics are reported as mean ± standard deviation (SD). Comparison between the groups are calculated by Mann–Whitney testing or one-way ANOVA followed by Dunnett’s multiple comparison post-test for continuous data and Fisher’s exact test for categorical data.

Antidiabetic regimen was similar in the groups A1A1 and AxA2: Prevalence of a combination therapy with oral antidiabetics and insulin was 9% in A1A1 and 11% in AxA2, insulin therapy was more often in A1A1 (13%) than in AxA2 (11%), 40% of A1A2 participants received oral antidiabetics only, whereas in the AxA2 group this therapeutic regimen was followed by 38%, the remaining participants in the groups did not receive any antidiabetic medication. Participants with diabetes significantly received more often acetylsalicyl acid than non-diabetics (25.0% vs 5.3%), but the application of acetylsalicyl acid did not differ between the groups A1A1 and AxA2. Regarding antidiabetic and antithrombotic therapy a possible confounding between the groups A1A2 and AxA2 therefore is not to be expected.

Mean diabetes duration did not differ statistically significant in both groups (A1A1 and AxA2) (9.3 ± 8.1 years vs. 9.5 ± 7.9 years, respectively).

### PLA1A2 genotype association with HbA_1c_ and MPV

An association of PLA1A2 genotype with diabetes mellitus type 2 was suggested from our previous publication [23]. Taking into account only individuals with diabetes (diagnosed diabetes or HbA_1c_ ≥6.5% (48 mmol/mol), N = 209), an association between PLA1A2 and elevated HbA_1c_ levels could be found (Table 2). However, no significant association between HbA_1c_ and genotype AxA2 was found regarding the whole cohort (4,028 participants) after multivariable adjustment (Table 2).

**Table 2:** **HbA**_**1c **_**level and platelet morphology in the participants according to diabetic state and PLA1A2 polymorphism**

	**Values**		**Logistic regression**	
**All (N = 4,028)**	**A1A1 (mean ± SD)**	**AxA2 (mean ± SD)**	**Odds ratio (95% CI)**	***P***
HbA_1c_ [%]	5.56 ± 0.58	5.60 ± 0.71	1.03 (0.96, 1.11)	0.42
HbA_1c_ [mmol/mol]	37.3 ± 6.3	37.7 ± 7.8		
MPV [fl]	8.76 ± 0.96	8.66 ± 0.89	0.90 (0.83, 0.96)	0.003
Platelet mass	2,100 · 2 ± 454.4	2,090.4 ± 460.8	0.99 (0.92, 1.07)	0.75
Platelet count [nl]	242.7 ± 58.3	243.9 ± 57.4	1.03 (0.96, 1.12)	0.33
**Diabetes (N = 209)**				
HbA_1c_ [%]	7.04 ± 1.41	7.63 ± 1.64	1.19 (1.04, 1.35)	0.01
HbA_1c_ [mmol/mol]	53.4 ± 15.4	68.9 ± 8.9		
MPV [fl]	9.02 ± 1.07	8.97 ± 0.96	1.03 (0.76, 1.38)	0.86
Platelet mass	1,996.80 ± 516.49	1,979.13 ± 420.85	0.93 (0.67, 1.30)	0.70
Platelet count [nl]	224.97 ± 65.86	223.20 ± 52.06	0.91 (0.66, 1.25)	0.57
**Non-Diabetes (N = 3,819)**				
HbA_1c_ [%]	5.48 ± 0.35	5.48 ± 0.36	0.97 (0.85, 1.11)	0.69
HbA_1c_ [mmol/mol]	36.4 ± 3.8	36.4 ± 3.9		
MPV [fl]	8.74 ± 0.95	8.64 ± 0.89	0.89 (0.83, 0.96)	0.002
Platelet mass	2,105.76 ± 450.22	2,096.83 ± 462.37	0.99 (0.91, 1.07)	0.85
Platelet count [nl]	243.61 ± 57.72	245.11 ± 57.46	1.04 (0.97, 1.12)	0.26

The estimates are shown as the adjusted odds ratio with 95% confidence interval (CI) for every unit increase of HbA_1c_. Model was adjusted for age, sex, waist-hip ratio, blood pressure (diastolic and systolic), cholesterol (total, HDL, LDL), smoking status (categorized: non-smoker, former smoker, current smoker), alcohol intake categorized: ≥20 g/day for women; ≥40 g/day for men), physical activity (categorized: >1 h per week).

A significant association between genotype AxA2 and lower MPV was demonstrated in the whole cohort and the group of non-diabetic people, but there was no significant association for platelet mass and platelet count (Table 2). Furthermore, a strong correlation of MPV with platelet count (r = -0.42, p < 0.0001) was detected. No significant correlation of MPV and platelet mass (r = 0.01, p = 0.53) was found. Age (β = -0.003, p = 0.015), HbA_1c_ (β = 0.108, p < 0.0001), BMI (β = 0.023, p < 0.0001), waist-hip-ratio (β = -1.816, p < 0.0001) were significantly associated with MPV in multivariate linear regression model.

### PLA1A2 genotype association with all-cause mortality

Within 10 years 239 (5.9%) patients died: 167 (5.7%) in the A1A1, 72 (6.5%) in the AxA2 (68 (6.6%) in the A1A2 and 4 (4.5%) in the A2A2) group, mainly due to cardiovascular diseases like myocardial infarction and ischemic heart disease (49%) as well as cancer (39%). No significant impact of genotype or HbA_1c_ on overall death was found (AxA2: adjusted HR (95% CI) =1.17 (0.59, 2.32); HbA_1c_: adjusted HR (95% CI) =1.05 (0.83, 1.33)) regarding all participants.

### HbA_1c_ association with all-cause mortality

Investigating the prediction of all cause of death by HbA_1c_, genotype and platelet morphology (MPV, platelet count, and platelet mass) in the non-diabetic participants (HbA_1c_ < 6.5% (48 mmol/mol)), we confirmed HbA_1c_ = 5.5% (37 mmol/mol), which was reported by Kowall et al. regarding KORA S4 participants aged from 55 to 75 years [28], as a cut-off for positive correlation between HbA_1c_ level and risk of all-cause mortality, but PLA1A2 polymorphism was not significantly associated with overall cause of death in this group (Table 3). HbA_1c_ was a strong predictor of survival in non-diabetic subjects with HbA_1c_ > 5.5% (37 mmol/mol), but was not related to survival in subjects with HbA_1c_ ≤ 5.5% (37 mmol/mol) (Table 3). However, in participants with HbA_1c_ ≤ 5.5% (37 mmol/mol) platelet count and platelet mass, but not MPV were associated with all-cause mortality (Table 3).

**Table 3:** **Association of mortality with genotype, HbA**_**1c **_**and platelet morphology in non-diabetic participants**

	**HbA**_**1c **_**≤5.5%**		**5.5% < HbA**_**1c **_**< 6.5%**	
	**(HbA**_**1c **_**≤37 mmol/mol)**		**(37 mmol/mol < HbA**_**1c **_**< 48 mmol/mol)**	
	**(N = 2,192)**		**(N = 1,627)**	
	**HR (95% CI)**	**P**	**HR (95% CI)**	**P**
Genotype AxA2	0.87 (0.54, 1.41)	0.57	0.99 (0.66, 1.53)	0.97
HbA_1c_	0.67 (0.24, 1.83)	0.43	2.47 (1.04, 5.88)	0.04
Platelet count	1.03 (1.00, 1.07)	0.03	0.99 (0.96, 1.02)	0.67
Platelet mass	1.00 (0.99, 1.00)	0.03	1.00 (0.995, 1.003)	0.95
MPV	2.13 (0.98, 4.60)	0.06	1.03 (0.51, 2.08)	0.93

The hazard ratios (HR) with 95% confidence interval (CI) for every unit increase of the variables are shown. Multivariate analysis of the association between mortality and genotype, HbA_1c_ and platelet markers, were taken with adjustment of age, sex, waist-hip ratio, blood pressure (diastolic and systolic), cholesterol (total, HDL, LDL), smoking status (categorized: non-smoker, former smoker, current smoker), alcohol intake (categorized: ≥20 g/day for women; ≥40 g/day for men), physical activity (categorized: >1 h per week).

We further analyzed the association of mortality with HbA_1c_ and platelet morphology (MPV, platelet count, and platelet mass) in separated genotypes to investigate possible genotype-specific effects (Table 4). In non-diabetic participants HbA_1c_ > 5.5% (37 mmol/mol) up to <6.5% (48 mmol/mol) a significant positive relation was found between HbA_1c_ level and all-cause mortality only in people with AxA2 genotype (Table 4), pointing to a combined effect of HbA_1c_ and AxA2 genotype in this subgroup. For the participants with HbA_1c_ ≤ 5.5% (37 mmol/mol), HbA_1c_ was not significantly associated with mortality (Table 4). Non-significant negative associations of HbA_1c_ level and risk of mortality were found in both genotype groups, which might imply higher risk of mortality in subjects with low level of HbA_1c_ (HbA_1c_ < 5.5% (37 mmol/mol)) (Table 4). In participants with genotype A1A1 and HbA_1c_ < 5.5% (37 mmol/mol), platelet count, platelet mass and MPV were associated with the all-cause mortality (Table 4).

**Table 4:** **Association of mortality with HbA**_**1c **_**and platelet morphology in non-diabetic participants according to PLA1A2 polymorphism**

	**HbA**_**1c **_**≤5.5%**				**5.5% < HbA**_**1c **_**< 6.5%**			
	**(HbA**_**1c **_**≤37 mmol/mol)**				**(37 mmol/mol < HbA**_**1c **_**< 48 mmol/mol)**			
	**A1A1(N = 1,597)**		**AxA2(N = 595)**		**A1A1(N = 1,167)**		**AxA2(N = 460)**	
	**HR (95% CI)**	***P***	**HR (95% CI)**	***P***	**HR (95% CI)**	***P***	**HR (95% CI)**	***P***
HbA_1c_	0.81 (0.23, 2.82)	0.74	0.52 (0.08, 3.31)	0.49	1.49 (0.50, 4.49)	0.48	7.85 (1.66, 37.14)	0.009
Platelet count	1.04 (1.00, 1.07)	0.03	1.02 (0.94, 1.10)	0.69	0.98 (0.95, 1.02)	0.35	1.01 (0.95, 1.08)	0.65
Platelet mass	0.996 (0.992, 0.999)	0.03	1.00 (0.94, 1.10)	0.66	1.00 (0.997, 1.005)	0.60	1.00 (0.991, 1.004)	0.52
MPV	2.46 (1.07, 5.61)	0.03	1.34 (0.18, 9.81)	0.77	0.78 (0.33, 1.85)	0.58	1.85 (0.44, 7.76)	0.40

The estimate indicates the hazard ratio (HR) with 95% confidence interval (CI) for every unit increase of the variable. The model was adjusted for age, sex, waist-hip ratio, blood pressure (diastolic and systolic), cholesterol (total, HDL, LDL), smoking status (categorized: non-smoker, former smoker, current smoker), alcohol intake (categorized: ≥20 g/day for women; ≥40 g/day for men), physical activity (categorized: >1 h per week).

**Figure 1:**
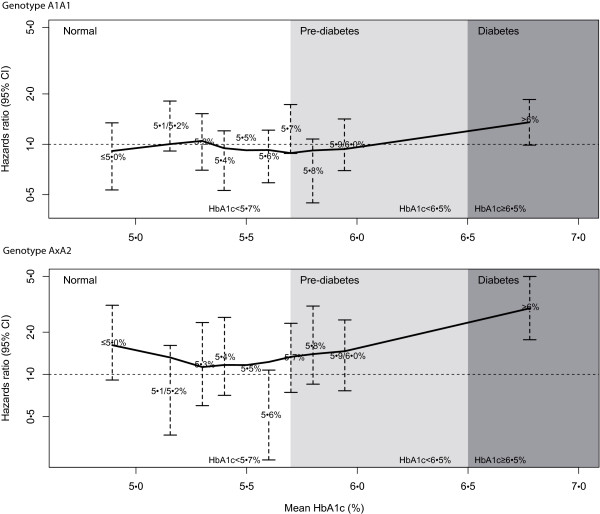
**Multivariate adjusted hazards ratio of mortality by category of HbA**_**1c **_**level in people with different genotypes.** Definition of pre-diabetes and diabetes follows definition criteria of the American Diabetes Association (ADA) (20). The hazard ratios and 95% confidence interval are given for each decile of HbA_1c_ level (≤5% (31 mmol/mol), 5.1% (32 mmol/mol)/5.2% (33 mmol/mol), 5.3% (34 mmol/mol), 5.4% (36 mmol/mol), 5.5% (37 mmol/mol), 5.6% (38 mmol/mol), 5.7% (39 mmol/mol), 5.8% (40 mmol/mol), 5.9% (41 mmol/mol)/6.0% (42 mmol/mol), >6.5% (48 mmol/mol)), taking HbA_1c_ = 5.5% (37 mmol/mol) as the reference. The position of the points on the x-axis represents the median value of baseline HbA_1c_ in the decile. The model was adjusted for age, sex, waist-hip ratio, RR diastolic, RR systolic, total cholesterol, HDL cholesterol, LDL cholesterol, smoking status (categorized: non-smoker, former smoker, current smoker), alcohol intake (categorized: ≥20 g/day for women; ≥40 g/day for men), physical activity (categorized: >1 h per week), platelet mass, mean plate volume and platelet count.

The hazard ratios of all-cause mortality in each decile of HbA_1c_ level in all participants with different genotypes, taking HbA_1c_ = 5.5% (37 mmol/mol) as reference is presented in Figure 1, corresponding data are shown in Table 5. The participants with genotype AxA2 had a high increase rate in relative risk from HbA_1c_ = 5.5% (37 mmol/mol) to higher HbA_1c_, while in participants with genotype A1A1, the increase was much less pronounced. In the highest decile (HbA_1c_ > 6.0% (42 mmol/mol)), the adjusted hazard ratio (95% CI) was 2.97 (1.07, 8.24) in people with genotype AxA2 compared to 1.35 (0.73, 2.51) in people with A1A1.

**Table 5:** **Multivariate adjusted hazards ratio of mortality according to different categories of HbA**_**1c **_**level in people with different genotypes**

	**RR (95% CI)**	
**Categories of HbA1c level**	**Genotype: A1A1**	**Genotype: AxA2**
<5% (<31 mmol/mol)	0.85 (0.34, 2.09)	1.68 (0.51, 5.61)
5.1/5.2% (32/33 mmol/mol)	1.29 (0.66, 2.52)	0.77 (0.18, 3.27)
5.3% (34 mmol/mol)	1.03 (0.48, 2.21)	1.18 (0.31, 4.51)
5.4% (36 mmol/mol)	0.80 (0.36, 1.78)	1.34 (0.38, 4.72)
5.5% (37 mmol/mol)	1	1
5.6% (38 mmol/mol)	0.85 (0.42, 1.72)	0.51 (0.12, 2.18)
5.7% (39 mmol/mol)	1.23 (0.64, 2.39)	1.31 (0.43, 3.99)
5.8% (40 mmol/mol)	0.69 (0.29, 1.64)	1.62 (0.46, 5.69)
5.9/6.0% (41/42 mmol/mol)	0.99 (0.49, 1.99)	1.37 (0.44, 4.28)
>6.5% (>48 mmol/mol)	1.35 (0.73, 2.51)	2.97 (1.07, 8.24)

The hazard ratios and 95% confidence interval are given for each decile of HbA_1c_ level (≤5% (31 mmol/mol), 5.1% (32 mmol/mol)/5.2% (33 mmol/mol), 5.3% (34 mmol/mol), 5.4% (36 mmol/mol), 5.5% (37 mmol/mol), 5.6% (38 mmol/mol), 5.7% (39 mmol/mol), 5.8% (40 mmol/mol), 5.9% (41 mmol/mol)/6.0% (42 mmol/mol), >6.5% (48 mmol/mol)), taking HbA_1c_ = 5.5% (37 mmol/mol) as the reference. The model was adjusted for blood pressure (diastolic and systolic), cholesterol (total, HDL, LDL), smoking status (categorized: non-smoker, former smoker, current smoker), alcohol intake (categorized: ≥20 g/day for women; ≥40 g/day for men), physical activity (categorized: >1 h per week), platelet mass, mean plate volume and platelet count.

## Discussion

Atherosclerosis results from complex interactions between the environment and genetic factors. Individual hemostatic platelet response may be influenced by the genetic profile of the platelet membrane glycoprotein (GP) receptors. As part of von Willebrand factor and fibrinogen receptor GPIIIa plays a pivotal role in platelet aggregation. Numerous polymorphisms in platelet surface glycoproteins have received particular interest [29]; one of those being the PLA1A2 polymorphism resulting in an exchange at the amino terminus of the ß3 subunit of the platelet fibrinogen receptor glycoprotein GPIIb/IIIa. Furthermore, the PLA2 allele has been associated with resistance to the antiplatelet agent such as aspirin [17]. Several lifestyle parameters like cholesterol-levels, physical activity, smoking habits and intake of alcohol were included in our analysis model to take into consideration the confounding potential on platelet morphology as described by Monteiro et al. [30]. In both groups, A1A1 as well as AxA2, hypoglycemic therapy was comparable, therefore, effects of antidiabetic medication on platelet morphology can be neglected. The analysis of the KORA cohort revealed no association between the PLA1A2 polymorphism and all-cause mortality and is thus confirmatory to the studies involving larger subject numbers. Our analysis revealed a non-linear relationship between HbA_1c_ and mortality in non-diabetic subjects and confirms previously published results regarding older participants of KORA S4.

### PLA2 and its relationsship to vascular outcome

The role of GPIIIa polymorphism in genetic susceptibility to clinical thrombotic disease still remains controversial [18,19]. The results of case–control association studies point to different directions, even within the same ethnic groups, and the association was hardly found in studies with larger sample size [18,31]. Whether there is an association between PLA-polymorphism and survival or cardiovascular outcome cannot be answered finally. Current available genome wide association studies (GWAS) data on platelet reactivity were recently summarized by Kuniki and Nugent [32]. Mesinger et al. evaluated in the KORA F3 500 K study data generated by Affymetrix 500 K Gene Chip analysis and did a replication in the KORA S4 cohort, which is topic of this publication. They found in KORA F3 3 common SNPs being strongly associated with MPV, but PLA1A2 was not among these [33]. Following a functional genomics approach no association was found between PLA1A2 polymorphism and platelet response parameters which might be caused by a low minor allele frequency (MAF) of 0.15 of the SNP and the studies conducted so far being underpowered to show effects of SNPs with such low MAF [32,33]. In another GWAS analysis done by Weiss et al. the ITGB3 polymorphism was identified as a quantitative trait locus (QTL) for whole blood serotonin. Whether this is of impact for platelet activation and platelet function has not yet been examined [34].

### PLA2 and its role in diabetes mellitus and prediabetes

Our data do not suggest an overall linkage between PLA1A2 genotype and all-cause mortality but put further substance to the assumption that the PLA2 SNP is associated with impaired metabolic control in diabetes mellitus. In the group of non-diabetics with higher HbA_1c_ (HbA_1c_ > 5.5% (37 mmol/mol) to <6.5% (48 mmol/mol)) including prediabetic subjects, people with PLA2 SNP showed higher mortality risk for same increase of HbA_1c_ level compared to persons with HbA_1c_ ≤ 5.5% (37 mmol/mol), pointing to a combined effect of HbA1c and AxA2 genotype in this subgroup. This effect was not detected in A1A1 subjects. HbA_1c_ had no significant influence on survival in the diabetic patients of KORA; this might be due to the limited number of subjects with elevated HbA_1c_ in this cohort.

HbA_1c_ turned out to be a strong predictor for survival in the non-diabetic population of the KORA cohort with the lowest risk at a baseline level of 5.5% (37 mmol/mol). This finding is in line with a previous publication by Kowall et al. regarding KORA S4 participants aged from 55 to 75 years [28]. Within the present analysis all participants of KORA S4, whose survival status and whose PLA1A2 genotype is known were included. Hyperglycemia is a strong predictor of mortality and cardiovascular risk and most of the observational studies show a linear positive association [35-37]. However, some other studies - like ours excluding manifest diabetics - suggest non-linear relationships (U- or J-shaped) between glycemic status and mortality risk [38-41]. These differences may rely on the characteristics of the study population and confounding factors (co-medication, diabetes duration, etc.). The KORA S4-cohort has a small number of diabetic subjects (N = 209; 5.2%) and a low overall mortality rate (5.9%). J- and U-shaped relationships of HbA_1c_ with overall mortality are seen in population studies without diabetes like the ARIC (Atherosclerosis Risk in Communities) study [39] and National Health and Nutrition Examination Survey (NHANES) [41] pointing to the possible effects of comorbidities and age or other yet unknown confounders, which might be similar to the KORA S4-cohort. A study involving diabetes patients showed U-shaped mortality curves with increasing HbA_1c_ values and optimal HbA_1c_ with lowest mortality events around 7.5% [40]; the higher mortality associated with low HbA_1c_ may be attributable to the level of comorbidities [42].

Our results may imply that non-diabetic people with AxA2 are more sensitive to the level of HbA_1c_, and suffer higher relative risk of mortality than people with A1A1 genotype for the same increase of HbA_1c_ level in the range of 5.5% (37 mmol/mol) to 6.5% (48 mmol/mol), resembling a prediabetic metabolic situation. The association of HbA_1c_ and all-cause of death was PLA1A2 genotype specific. At each decile the curve of AxA2 carriers is shifted to elevated hazard ratios, while in people with A1A1 the HbA_1c_ level was not significantly associated with mortality (Table 3). The curve presented in Figure 1 showing the hazards ratio for each HbA_1c_ decile documents the higher risk of AxA2 carriers with increasing HbA_1c_. In this special constellation mortality obviously seems to be driven by the more thrombogenic AxA2 allele.

### Platelet functional markers and the prediction of survival in healthy people

An important additional finding on survival prediction in our analysis is that MPV, platelet count and platelet mass are significantly associated with survival in the individuals with HbA_1c_ less than 5.5% (37 mmol/mol). This fact clearly points to the role of platelet characteristics in the group of definite non-diabetics, contributing to survival/mortality. In this cohort subjects with AxA2 genotype present with significant lower MPV but preserved platelet mass, which might be an indicator of increased platelet consumption, mainly of the larger platelet-subpopulation that present more receptors per platelet [23]. Due to slightly higher numbers in platelet count this effect may be compensated by keeping the platelet mass constant to sustain regular functionality. Besides elevated age, smoking, alcohol consumption, platelet morphology and mass may contribute to the higher mortality risk in these individuals.

## Conclusion

PLA2 significantly correlates with mortality in non-diabetics with HbA_1c_ values of >5.5% (37 mmol/mol) up to 6.5% (48 mmol/mol), including the prediabetic subjects. Therefore, even the prediabetic subject has to be regarded as a vulnerable vascular patient, which has recently been confirmed by the Silent Diabetes Study, published by Doerr and co-authors [43]. Elevated blood glucose levels beyond the diabetic threshold are a powerful predictor of 30 day mortality in acute heart failure patients, emphasizing the critical role of the prediabetic state [44]. Our results suggest the need for a graded interventional hierarchy supporting antiplatelet therapy in nondiabetics, maintenance of euglycemia and antiplatelet therapy in prediabetic AxA2 subjects whereas in manifest diabetes euglycemia is recommended as the most important therapeutic aim.

## Abbreviations

KORA: Kooperative Gesundheitsforschung im Raum Augsburg (Cooperative Health Research in the Region of Augsburg); GP: Glycoprotein; GWAS: Genome wide association studies; MPV: Mean platelet volume; FBG: Fasting blood glucose; QTL: Quantitative trait locus; MAF: Minor allele frequency.

## Competing interests

The authors declare that they have no competing interests.

## Authors’ contributions

All authors had access to the final data, the manuscript and accept the responsibility for its validity. The authors declare that there is no conflict of interest. BS literature search, data analysis, data interpretation, writing of the manuscript. TX data analysis, data interpretation, writing of the manuscript, design of figures. CM data collection, data interpretation, BM data collection, CH data collection, MR data handling, HG data collection and handling. AP data analysis, design of figures. WK data collection. TI data collection and handling. HW data collection. RWS data analysis. WR data collection. DT data collection, data interpretation, writing of the manuscript. All authors had access to the final data, the manuscript and accept the responsibility for its validity.
